# Omitted staging PSMA PET/CT is associated with advance disease extent at time of PSA persistence: a single center retrospective analysis

**DOI:** 10.1007/s00259-026-07799-1

**Published:** 2026-03-10

**Authors:** Andrea Di Giorgio, Marco Rapa, Martina Di Franco, Caterina Maria Paola Sgro, Giambattista Siepe, Veronica Mollica, Lorenzo Bianchi, Matteo Droghetti, Paolo Castellucci, Riccardo Mei, Andrea Farolfi, Riccardo Schiavina, Stefano Fanti

**Affiliations:** 1 Nuclear Medicine, Alma Mater Studiorum, University of Bologna, Via Massarenti 9, Bologna, 40138 Italy; 2 Radiation Oncology, IRCCS Azienda Ospedaliero-Universitaria di Bologna, Bologna, 40138 Italy; 3 Medical Oncology, IRCCS Azienda Ospedaliero-Universitaria di Bologna, Bologna, Italy; 4 Division of Urology, IRCCS Azienda Ospedaliero-Universitaria di Bologna, University of Bologna, Bologna, Italy; 5 Nuclear Medicine, IRCCS, Azienda Ospedaliero-Universitaria Di Bologna, Bologna, Italy

**Keywords:** PSMA PET/CT, Prostate Cancer, Persistence Disease, Staging, Intermediate Risk

## Abstract

**Introduction:**

PSMA PET has emerged as a crucial imaging modality for staging intermediate- and high-risk prostate cancer (PCa), yet its implementation varies globally. This study aims to assess the diagnostic consequence of omitting pre-operative PSMA PET/CT staging by comparing the radiological features and extent of disease in two cohorts of patients who subsequently developed PSA persistence (> 0.1 ng/mL) following radical prostatectomy (RP).

**Materials and methods:**

Single-center, retrospective study (October 2019 to May 2025). The patient cohort included individuals who underwent radical prostatectomy and subsequently experienced PSA persistence, defined as a PSA value ≥ 0.1 ng/ml at the first measurement after 4–8 weeks after RP. Patients were divided into two groups: a Staging Cohort who underwent a pre-operative [68Ga]Ga-PSMA-11 PET/CT and a Non-Staging Cohort who did not. All patients received a [68Ga]Ga-PSMA-11PET/CT scan for PSA persistence. Clinical data, including PSA, TNM stage, and ISUP grade, as well as follow-up data were collected. The primary endpoints were the comparison of the overall PSMA PET positivity rate and the extent of both locoregional (N) and distant metastatic (M1) disease between the two cohorts. Secondary analysis included a comparison of baseline pathological characteristics and the evaluation of the potential for a theoretical change in initial patient management.

**Results:**

147 patients with PSA persistence after RP were included and divided into the Staging Cohort (*n* = 72, 49%) and a Non-Staging Cohort (*n* = 75, 51%). Baseline data showed the Staging Cohort had a higher proportion of high-grade (ISUP 4/5:72% vs. 45%; *p* < 0.05 ) and locally advanced (T3/T4: 78% vs. 61%; *p* < 0.05) disease. The Non-Staging Cohort had a higher rate of positive [68Ga]Ga-PSMA-11 PET/CT scans for persistence (59%) compared to the Staging-Cohort (42%). The Non-Staging Cohort showed a markedly greater extent of disease, with a high proportion of patients (*n* = 34/75,45%) demonstrating findings consistent with M1 disease (bone or distant lymph nodes). The anatomical distribution of positive lymph node sites also differed: the Staging Cohort showed higher obturator nodes involvement (48% of positive sites), while the Non-Staging Cohort had a significantly higher proportion in common iliac (34%) and external/internal iliac sites. A retrospective evaluation of the Non-Staging Cohort suggested that a pre-operative [68Ga]Ga-PSMA-11 PET/CT would have triggered a theoretical change of management in all 44 positive cases, primarily by suggesting systemic therapy instead of RP for M1 patients.

**Conclusion:**

The omission of pre-operative [68Ga]Ga-PSMA-11 PET/CT is associated with a significantly higher incidence of PSMA-positive and extensive disease (locoregional and metastatic) at the time of PSA persistence. This suggests a critical diagnostic gap and potential under-staging in the Non-Staging Cohort, underscoring the vital role of early PSMA PET/CT utilization to optimize initial risk stratification and treatment planning.

## Introduction

Prostate cancer (PCa) remains one of the most frequently diagnosed cancers among men globally (1). Accurate pre-operative staging and risk stratification are critically important, as they dictate the choice of curative approach (2). Radical prostatectomy (RP) serves as one key definitive treatment option, but curative intent can also be achieved via definitive radiation therapy (RT), often combined with androgen deprivation therapy (ADT) for high-risk disease (3, 4). Identifying the extent of disease, particularly the presence of extra-prostatic extension or regional and distant metastases, is essential to guide initial management, determine the necessity of an extended lymph node dissection (eLND) during RP, or tailor the RT field and the intensity of systemic therapies. For decades, staging relied primarily on conventional imaging, namely computed tomography (CT) and bone scintigraphy (BS) (5). However, the limited sensitivity of these modalities, especially in detecting small-volume nodal metastases, frequently leads to under-staging in patients with intermediate- or high-risk features (6). This diagnostic shortfall means that patients with undetected microscopic or oligometastatic disease may undergo RP, which is then destined to fail shortly after surgery (7). The introduction of Prostate-Specific Membrane Antigen (PSMA) PET/CT has fundamentally shifted the staging paradigm. PET/CT with 68-gallium or 18-fluorine labelled PSMA ligands has demonstrated significantly superior sensitivity and specificity compared to conventional imaging, effectively identifying disease sites at lower Prostate-Specific Antigen (PSA) levels and altering treatment plans in a high percentage of patients (8). Consequently, international guidelines increasingly recommend its use for initial staging in intermediate-unfavorable and high-risk PCa (9, 10). Despite the compelling evidence, the real-world utilization of pre-operative PSMA PET/CT remains heterogeneous. A direct clinical challenge that often follows RP is PSA persistence, defined as a non-normalized PSA value (typically ≥ 0.1 ng/mL) immediately following surgery (11). While PSA persistence itself is a multifactorial phenomenon—potentially arising from incomplete surgical resection, low PSMA expression disease, or microscopic tumor burden missed by even molecular imaging—it serves as a critical, early indicator of treatment failure (12, 13). While recent clinical investigations focus on the timing and prognostic implications of the level of persistent PSA, the contribution of the reliance on conventional imaging (omitted PSMA PET/CT) to the nature and anatomical extent of the persistent disease remains largely unquantified (14, 15). We hypothesize that the omission of pre-operative [68Ga]Ga-PSMA PET/CT leads to an initial under-staging, which subsequently results in the manifestation of more diffuse and extensive locoregional and distant disease at the time of PSA persistence. Therefore, this retrospective cohort study aims to assess this under-staging gap by comparing the PSMA PET/CT positivity rates, and the incidence and anatomical distribution of both advanced locoregional (N) and distant metastatic (M1) disease identified during the workup for PSA persistence in patients who received pre-operative [68Ga]Ga-PSMA PET/CT versus those who did not, highlighting the implications for a theoretical change in initial therapeutic management.

## Materials and methods

### Study design and patient population

This is a single-center, retrospective study conducted from October 2019 to May 2025. The study cohort was comprised of patients who underwent RP for PCa, subsequently experienced PSA persistence, and were referred for a [68Ga]Ga-PSMA-11 PET/CT. For the purpose of this study, PSA persistence was strictly defined as two consecutive PSA value ≥ 0.1 ng/mL at the first measurement 4–8 weeks after radical prostatectomy. Patients were included if complete pathological data from the surgical specimen were available. Exclusion criteria included a lack of histological data, diagnosis of neuroendocrine tumor, or prior exposure to systemic therapies (e.g., neoadjuvant or adjuvant hormonal therapy or chemotherapy) before the PSMA PET/CT scan for persistence. All patients who met the inclusion criteria provided written informed consent. The study was approved by the institutional ethics committee as part of a comprehensive basket protocol (Ref. 244/2016/O/Oss—8 November 2016) and was conducted in accordance with the Declaration of Helsinki.

The final cohort was dichotomized into two distinct groups based on their initial diagnostic pathway: the Staging Cohort, who underwent a [68Ga]Ga-PSMA-11 for pre-operative staging, and the Non-Staging Cohort, who did not receive a staging PET prior to primary treatment.

### Image acquisition and analysis

All PSMA PET/CT scans were performed using a standard institutional protocol. The radiotracer utilized was [68Ga]Ga-PSMA-11, administered as an intravenous bolus. Images were acquired with a standard uptake time of 60 ± 10 min post-injection. PSMA PET findings were interpreted independently by three experienced nuclear medicine physicians, with discrepancies resolved by consensus. The interpretation of the scans was based on a combination of visual analysis and established criteria, including the EAU-ESTRO guidelines and the PROMISE criteria version 2 (16).

### Clinical and pathological data collection

Clinical and pathological data were collected from the electronic medical records. The collected variables included pre-operative PSA and PSA values at the time of the PET for persistence. Pathological variables (TNM stage and ISUP Grade Group) were derived exclusively from the definitive post-operative surgical specimen report.

### Study endpoints

Our primary endpoints were two-fold: first, to compare the overall positivity rate observed during the workup for PSA persistence between the Staging Cohort and the Non-Staging Cohort. Second, to compare the extent of disease identified by the [68Ga]Ga-PSMA-11 PET/CT for persistence, specifically the incidence and anatomical distribution of both N and M1 findings between the two groups according to miTNM (12). 

The secondary endpoints included comparing the baseline demographic and pathological characteristics (e.g., ISUP Grade, T-Stage) of the two cohorts. Moreover, we aimed to analyze the frequency of positive [68Ga]Ga-PSMA-11 PET/CT findings for persistence in the Non-Staging Cohort and evaluating a theoretical change of management. The theoretical change of management was assessed by having a multidisciplinary expert panel (consisting of a radiation oncologist, urologist, and medical oncologist) independently review the [68Ga]Ga-PSMA-11 PET/CT findings. A change was defined as the panel’s consensus that the pre-operative identification of M1 disease would have led to potential systemic therapy instead of curative-intent treatment, or extensive locoregional disease would have prompted different primary local treatment (e.g., extended lymphadenectomy or RT field adjustment). Furthermore, we aimed to analyze the frequency of positive [68Ga]Ga-PSMA-11 PET/CT findings for persistence within the low- and intermediate-risk subgroups (ISUP Grade Groups 1 and 2) of the Non-Staging Cohort, where pre-operative imaging is not indicated based on current guidelines.

### Statistical analysis

Descriptive statistics were used to summarize the patient and tumor characteristics. Continuous variables (e.g., age and PSA levels) were presented as the median and interquartile range (IQR). Comparisons between the Staging Cohort and the Non-Staging Cohort were performed using the two-sided independent t-test for normally distributed data or the Mann-Whitney U test for non-normally distributed data. Categorical variables (e.g., ISUP Grade Group, pathological TNM stage, and positivity) were summarized using counts and percentages and compared using the chi-squared test (x^2^) or Fisher’s exact test when appropriate. A p-value of less than 0.05 was considered statistically significant. All statistical analyses were conducted using R software (version 4.5.1).

## Results

### Patient cohort and baseline characteristics

A total of 147 patients with PSA persistence following radical prostatectomy were included in this retrospective analysis. The final cohort was clearly delineated into the Staging Cohort (*n* = 72, 49%), who received pre-operative [68Ga]Ga-PSMA-11 PET/CT, and the Non-Staging Cohort (*n* = 75, 51%), who did not (Table 1). The absence of pre-operative PSMA PET in the Non-Staging Cohort was attributed to two primary factors: its non-indication based on standard guidelines at the time of initial diagnosis for patients classified as favorable-intermediate risk, or logistical barriers, including limited local availability. Pre-operative Multiparametric Prostate Magnetic Resonance Imaging (mpMRI) data were analyzed for 99 patients (67% of the total cohort). This analysis found no statistically significant difference in high-risk PIRADS scores (4/5) between the Staging and Non-Staging Cohorts (*p* = 0.291). Initial staging in the Non-Staging Cohort (*n* = 75) relied exclusively on conventional imaging. Specifically, 56 patients (75%) underwent a pre-operative BS, and 69 patients (92%) underwent a pre-operative CT scan, and 51 patients (68%) received both BS and CT scan. All bone scans were negative for metastasis. The analysis of cTNM data derived from CT, highlighted locally advanced disease (cT3a/b) in 28 (31%) of cases, with only 2 patients (3%) classified as cN1 (Table 2). Conversely, the Staging Cohort primarily used [68Ga]Ga-PSMA-11 PET/CT, with only a small minority utilizing also conventional imaging (*n* = 4, 6% both BS and CT scan). The median administered activity of [68Ga]Ga-PSMA-11 was 145Mbq (IQR 134–153) with a standard uptake time of 64 min (IQR 60 -72.5).

**Table 1:** Baseline Patient and Tumor Characteristics

Characteristic	*Total Cohort (n = 147)*	*Staging Cohort (n = 72)*	*Non-Staging Cohort (n = 75)*	*p*-value
Age (years)	67 (62–72)	67 (63–71)	66 (62–72)	0.254
Time from surgery to PET (months)	3 (2–5)	3 (2–5)	3 (2–6)	0.257
PSA at surgery (ng/mL)	11.4 (7-17.2)	11.9 (7.8–19.3)	10.5 (6.4–14)	0.398
PSA post-surgery (ng/mL)	0.5 (0.3–1.5)	0.5 (0.2–1.5)	0.5 (0.3–1.5)	0.172
*mpMRI Execution Status*,* n (%)*				
Performed	99 (67%)	57 (79%)	42 (56%)	0.003
Not Performed	3 (2%)	3 (4%)	0 (0%)	
Unknown Data	45 (31%)	12 (17%)	33 (44%)	
*PIRADS Available*,* n*	95	53	42	
PIRADS 2/3	11 (12%)	4 (8%)	7 (17%)	0.291
PIRADS 4/5	84 (88%)	49 (92%)	35 (83%)	0.291
mpMRI N1 (Lymph Nodes)	2 (2%)	2 (4%)	0 (0%)	0.614
*ISUP Grade*,* n (%)*				
ISUP 1	2 (1%)	1 (1%)	1 (1%)	1.000
ISUP 2	17 (12%)	3 (4%)	14 (19%)	0.009
ISUP 3	37 (25%)	12 (17%)	25 (33%)	0.023
ISUP 4	41 (28%)	27 (38%)	14 (19%)	0.016
ISUP 5	45 (31%)	25 (35%)	20 (27%)	0.371
ISUP Unknown	5 (3%)	4 (6%)	1 (1%)	0.203
*T-Stage*,* n (%)*				
T2	38 (26%)	16 (22%)	22 (29%)	0.351
T3a	58 (40%)	34 (47%)	24 (32%)	0.065
T3b	43 (29%)	21 (29%)	22 (29%)	1.000
T4	1 (1%)	1 (1%)	0 (0%)	0.489
N-Stage, n (%)				
N0	78 (53%)	46 (64%)	57 (76%)	0.149
N1	44 (30%)	26 (36%)	18 (24%)	0.149
Nx	16 (11%)	0 (0%)	16 (21%)	< 0.001
R-margin status, n (%)				
R0	85 (58%)	33 (46%)	52 (69%)	0.005
R1	62 (42%)	33 (46%)	29 (39%)	0.407

Continuous data are median and interquartile range. For categorical pathological variables (e.g., ISUP Grade, PIRADS score, T-stage), the p-value was calculated using Fisher’s Exact Test, while continuous demographic data (e.g., age, time from surgery, PSA values) were compared using the Mann-Whitney U test

As detailed in Table 1, demographic and temporal characteristics, such as median age and time elapsed from RP to the PSMA PET for persistence, showed no significant differences between the two cohorts (*p* > 0.05). However, a significant disparity in initial risk profiles was evident, reflective of the clinical selection criteria for advanced imaging. The Staging Cohort showed a significantly higher proportion of adverse pathological features: 52 patients (72%) were classified as high-grade (ISUP 4 or 5) compared to 34 patients (45%) in the Non-Staging Cohort (*p* < 0.05). Similarly, the extent of local disease was greater in the Staging Cohort, with 56 patients (78%) classified as T3a/T3b/T4, versus 46 patients (61%) in the Non-Staging Cohort (*p* < 0.05).

### PSMA PET positivity and extent of disease at persistence

Overall, the [68Ga]Ga-PSMA-11 PET/CT scan performed for PSA persistence demonstrated positive findings in 74 patients (50%) across the entire cohort. The Non-Staging Cohort yielded a significantly higher rate of positive PSMA PET/CT scans for persistence (44/75, 59%) compared to the Staging Cohort (30/72, 42%; *p* < 0.05).

To account for the influence of tumor stage on outcomes, a subgroup analysis was performed restricted to patients with locally advanced disease (pT3/pT4). In the Non-Staging Cohort, 27 out of 46 patients (59%) with pT3/pT4 disease had a positive PET, compared to 24 out of 56 patients (43%) in the Staging Cohort. Despite both cohorts sharing this same high-risk pathological stage, the rate of PET positivity for persistence differed, although this difference did not reach statistical significance (*p* = 0.16).

**Table 2:** Conventional Staging Modalities and Findings in the Non-Staging Cohort

Characteristic	Result *(Non-Staging Cohort*,* n = 75)*
Staging Techniques Used, n (%)	
CT Performed	69 (92%)
BS Performed	56 (75%)
BS and CT Combined	51 (68%)
Results of Conventional Staging	
BS Staging Result, n (%)	
Negative for Metastasis	56 (100%)
cTNM	69
cT2 Stage	25 (36%)
cT3a Stage	21 (31%)
cT3b Stage	7 (10%)
Unknown	16 (23%)
cN1	2 (3%)

The higher positivity translated to a greater extent of aggressive disease in the Non-Staging Cohort. Findings consistent with M1 disease (bone, visceral, common iliac, or extra-pelvic nodes) were identified in 34 patients (45%) of the Non-Staging Cohort, substantially exceeding the 21% (15 patients) found in the Staging Cohort. Overall, positive lymph node findings were observed in 55 patients (37%), with the Non-Staging Cohort having a higher absolute rate (34/75, 45%) compared to the Staging Cohort (21/72, 29%).

**Fig. 1:**
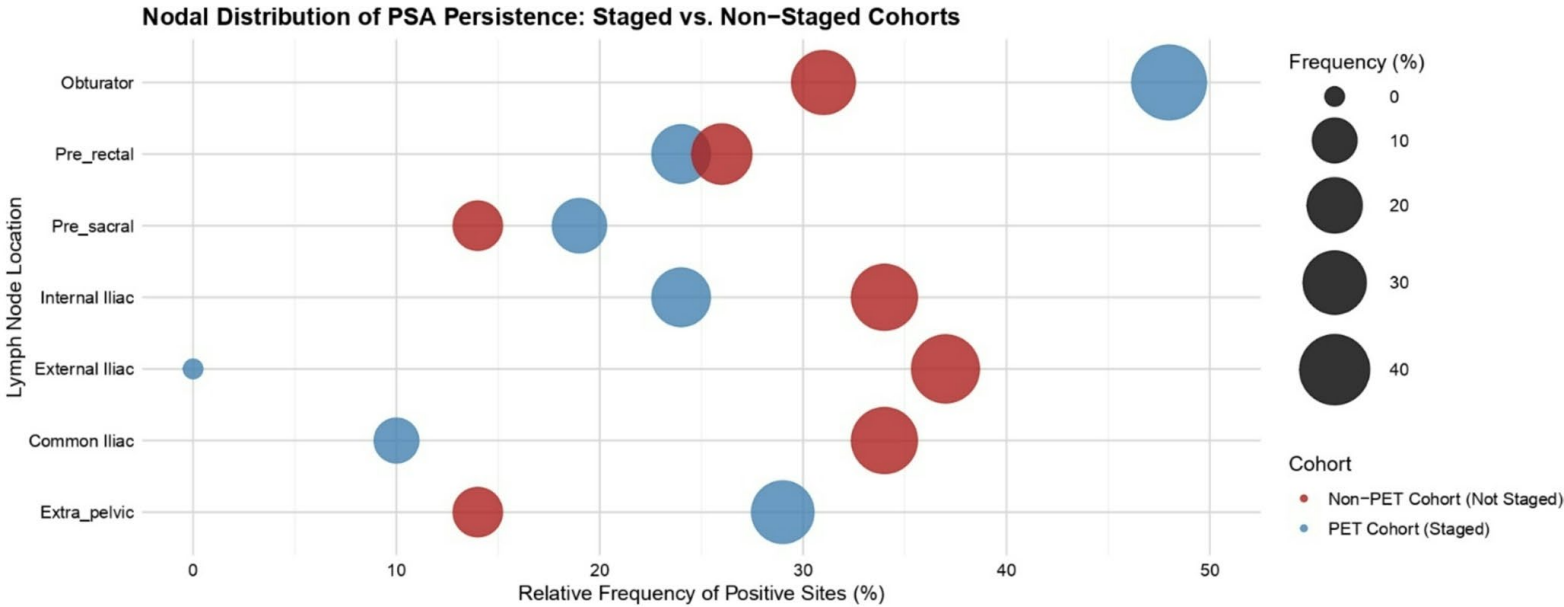
Nodal Distribution of PSA Persistence: Staged vs. Non-Staged Cohorts. This bubble plot compares the nodal distribution of PSA persistence across different lymph node locations between the Non-PET Cohort (red circles) and the PET Cohort (blue circles). The x-axis shows the Relative Frequency of Positive Sites (%) for each location within the respective cohort. The Frequency (%) of involvement for a specific lymph node site is represented by the size of the bubble

**Fig. 2:**
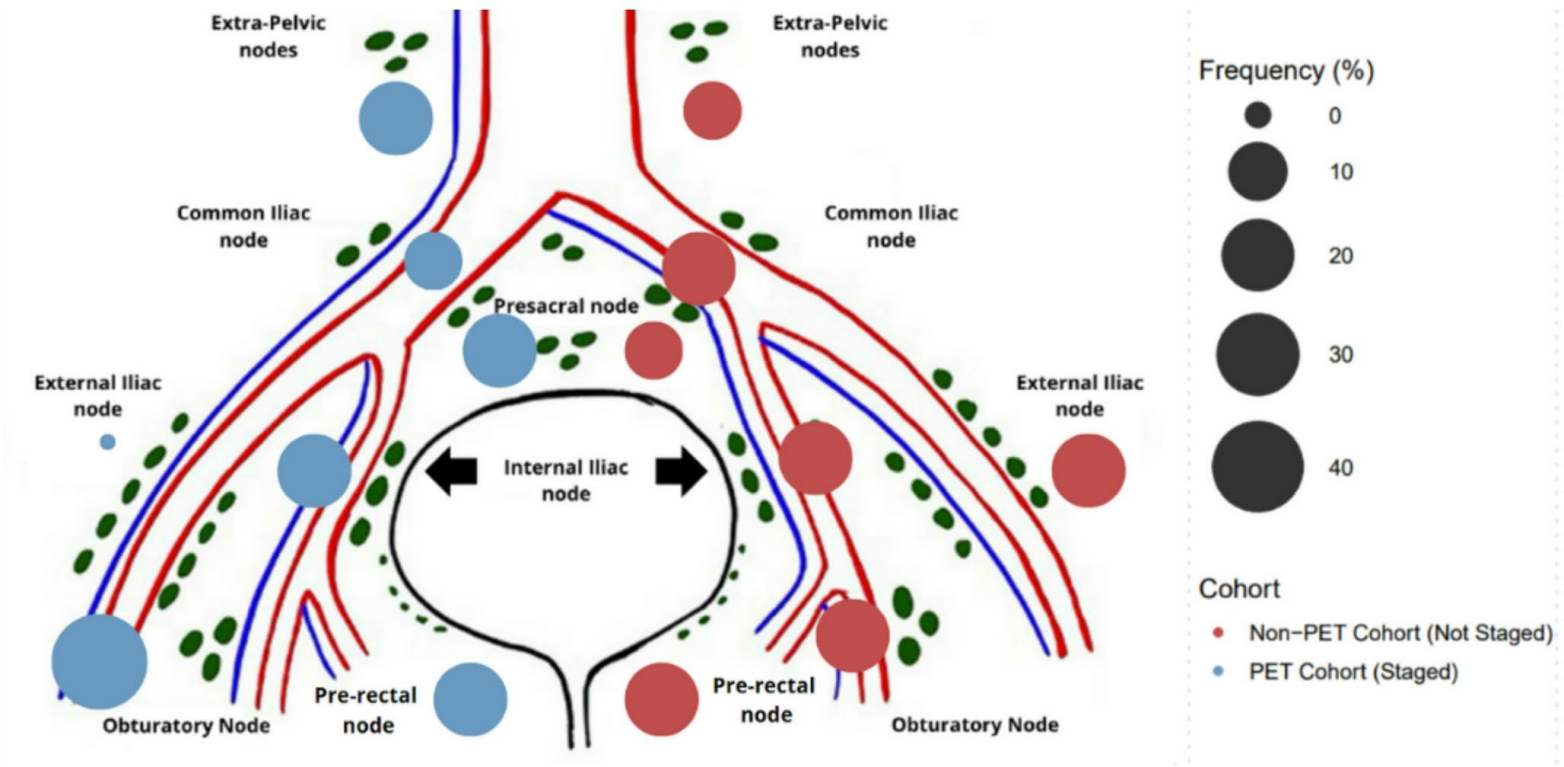
Anatomical schematic illustrating the Nodal Distribution of PSA Persistence: PET vs. Non-PET Cohorts. A schematic anatomical overlay showing the relative frequency and distribution of positive lymph node sites for the Non-PET Cohort (red circles) and the PET Cohort (blue circles). The size of the bubble indicates the Frequency (%) of involvement for that specific lymph node location, as shown in the legend. This schematic highlights the difference in common metastatic sites between the two staging strategies

Analysis of the anatomical distribution of positive lymph node sites further highlighted the difference in disease extent. While the Staging Cohort showed a higher proportion of positive sites in obturator nodes (48% of positive sites), suggesting more contained regional failure, the Non-Staging Cohort exhibited a significantly higher burden of advanced nodal involvement, including common iliac (34%) and external/internal iliac sites. Bone findings were observed in 32 patients (22%) across both cohorts, and visceral lesions in 2 patients (1%) (Table 3; Figs. 1 and 2).

**Table 3:** PSMA PET Findings for PSA Persistence

Findings, *n* (%)	*Total Cohort (n = 147)*	*Staging Cohort (n = 72)*	*Non-Staging Cohort (n = 75)*	*p*-value
Total PET positive	74 (50%)	30 (42%)	44 (59%)	0.048
PET positive - pT3/pT4	51 (35%)	24 (33%)	27 (36%)	0.16
*Positive Findings Location*,* n*	*Total PET positive (n = 74)*	*PET positive – PET Cohort (n = 72)*	*PET positive – Non-Staging Cohort (n = 72)*	
Prostatic Fossa	5 (7%)	1 (1%)	4 (6%)	0.366
Lymph nodes	55 (74%)	21 (29%)	34 (47%)	0.060
Bone	32 (43%)	15 (21%)	17 (24%)	0.843
Visceral	2 (3%)	1 (1%)	1 (1%)	1.000
*Lymph Node Location*,* n (%)*	*(n = 56)*	*(n = 21)*	*(n = 35)*	
External Iliac	13 (23%)	0 (0%)	13 (37%)	0.009
Internal Iliac	17 (30%)	5 (24%)	12 (34%)	0.551
Common Iliac	14 (25%)	2 (10%)	12 (34%)	0.056
Obturator	21 (38%)	10 (48%)	11 (31%)	0.263
Pre-rectal	14 (25%)	5 (24%)	9 (26%)	1.000
Pre-sacral	9 (16%)	4 (19%)	5 (14%)	0.715
Extra-pelvic	11 (20%)	6 (29%)	5 (14%)	0.297

p-value was calculated using Fisher’s Exact Test

### Subgroup analysis and clinical impact

Among the Non Staging cohort, 15/75 patients (20%) were low and favorable-intermediate risk PCa (ISUP 1–2), for whom pre-operative PSMA PET is not indicated based on current guidelines. Among this group, 4 out of 15 patients (27%) were found to have positive [68Ga]Ga-PSMA-11 PET/CT findings for persistence, totaling 7 lesions. All 4 patients had previously undergone both a pre-operative BS and a CT scan for initial staging. Notably, 3 out of 7 lesions were classified as M1 disease, specifically: 1 bone lesion in the left iliac wing and 2 common iliac lymph nodes. The remaining 4 lesions included 2 para-rectal, 1 obturator node and 1 external iliac lymph node. All these lesions were not detectable (bone) or interpreted as benign (sub centimetric lymph nodes) at conventional imaging.

**Fig. 3:**
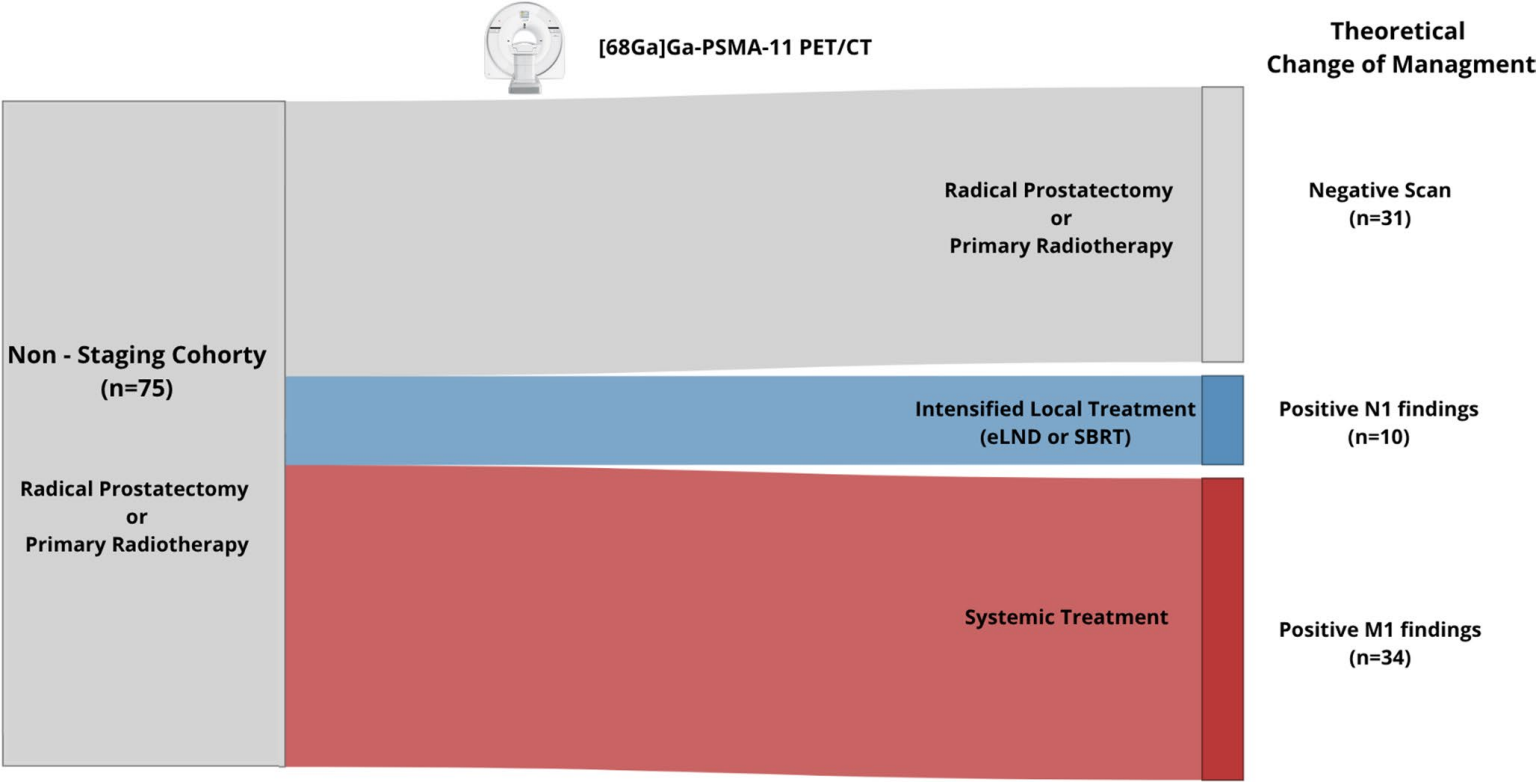
Theoretical Change of initial therapeutic management in the Non-Staging Cohort (*n* = 75) guided by [68Ga]Ga-PSMA-11 PET/CT findings. The Sankey diagram illustrates the proposed triage of patients in the Non-Staging Cohort based on the findings of the [68Ga]Ga-PSMA-11 PET/CT performed for PSA persistence. The width of the bands is proportional to the patient count (*n* = 75), demonstrating the profound clinical shift that molecular imaging would have enabled. The single node on the left represents the uniform actual treatment pathway received by all 75 patients (RP or primary RT) without PSMA PET/CT guidance. The nodes on the right represent the optimal, individualized pathways that would have been chosen had PSMA PET/CT findings been available pre-treatment. The flow reveals that 44 patients (59% of the cohort) should have been diverted to a different management strategy. Specifically, the 34 patients (77\% of the positives, shown in red) with M1 disease (osseous or common iliac lymph nodes) would have been managed with immediate Systemic Treatment, thereby avoiding inappropriate local therapy. The 10 patients (shown in blue) with locoregional disease would have prompted a change to Intensified Local Treatment (eLND or SBRT). Only the 31 patients with PSMA PET/CT-negative scans would have optimally remained on the initial RP or Primary RT pathway (shown in gray)

The clinical consequence of the diagnostic gap was demonstrated through a retrospective evaluation of the Non-Staging Cohort. A pre-operative PSMA PET/CT would have triggered a “theoretical change of management” in all 44 cases with a positive scan. Based on the positive findings, 34 patients (77% of positive cases) would have been immediate candidates for systemic therapy due to M1 disease (common iliac lymph node involvement: *n* = 12, 27% ; extra-pelvic lymph node involvement: *n* = 5, 11% ; and bone involvement: *n* = 17, 39%). The remaining 10 patients (23% of positive cases) with only pelvic lymph node involvement would have been candidates for extended lymphadenectomy or primary radiotherapy, representing a change in local management strategy (Figure 3).

## Discussion

This retrospective cohort study sought to evaluate the diagnostic consequences of omitting pre-operative PSMA PET/CT staging in patients who subsequently developed PSA persistence (PSA ≥ 0.1 ng/ml) following radical prostatectomy. Our primary finding supports the hypothesis that this omission results in a critical under-staging gap. Despite the Staging Cohort exhibiting a pathologically higher-risk profile at pre-operative assessment—a phenomenon consistent with established clinical criteria for advanced imaging utilization—the Non-Staging Cohort showed a higher overall [68Ga]Ga-PSMA-11 PET/CT positivity rate for persistence (59% vs. 42%; p-value 0.048).

This compelling difference is not incidental: it reflects the successful impact of optimal initial management guided by PSMA PET/CT. The use of molecular imaging likely induced a stage migration effect: patients identified pre-operatively with clinical nodal (cN1) or distant metastatic (M1) disease were likely shifted towards alternative treatment modalities, such as definitive radiotherapy or systemic therapy. Consequently, the patients in the Staging Cohort who proceeded to radical prostatectomy represented a highly selected population with a lower intrinsic risk of harboring residual or persistent disease compared to the unselected Non-Staging Cohort. Conversely, the high positivity rate in the Non-Staging Cohort suggests that the initial non-molecular staging (relying primarily on conventional CT/BS) in the Non-Staging Cohort failed to detect substantial disease burden that was nonetheless present. The patients in this cohort proceeded to RP with unrecognized extensive disease, which rapidly manifested as biochemical failure. Crucially, our data suggest that the diagnostic superiority of PSMA PET translates into tangible differences in disease control. A potential confounder in our analysis could have been the different pathological stages between the two cohorts. However, our subgroup analysis of patients with pT3/pT4 disease challenges this notion. Even when restricting the comparison to this uniform high-risk pathological category, the Non-Staging Cohort exhibited a trend toward a higher rate of residual disease compared to the Staging Cohort (59% vs 43%; p = 0.16). This finding implies that the absence of pre-operative molecular imaging led to under-treatment even in patients with locally advanced disease. Conversely, in the Staging Cohort, the pre-operative identification of disease likely guided a more tailored surgical approach (e.g., more extensive lymphadenectomy), thereby reducing the burden of residual disease detected at the time of persistence. The difference in PSMA PET positivity was not merely numerical; it reflected a greater metastatic burden of disease in the Non-Staging Cohort at the time of persistence. Specifically, 45% of the Non-Staging Cohort presented with findings consistent with M1 disease (including bone and advanced nodal involvement like common iliac nodes). This high incidence of distant or non-regional disease, identified after surgery, stands in stark contrast to the lower M1 rate in the Staging Cohort, where systemic disease was largely excluded pre-operatively. These findings are particularly relevant when considered the existing literature on intermediate-risk PCa staging. A systematic review by Bonaddio et al. on PSMA PET/CT in intermediate-risk PCa demonstrated a low overall pooled positivity rate of approximately 9% for detecting nodal or distant metastases (17). A separate work by Hartwieg et al. showed that positive staging findings were rare in patients who ultimately proceeded to RP, which highlights the low yield of conventional staging and bias toward negative imaging (18). Our study provides the critical missing link, we highlighted that the consequence of omitting advanced molecular staging in this heterogeneous group is not just a rarity of positive findings, but rather the failure to capture significant disease burden, leading to the rapid manifestation of extensive disease at biochemical persistence (59% positivity in the Non-Staging Cohort). This disparity suggests that staging favorable-intermediate risk patients with conventional imaging could be reconsidered. In our study the 27% failure rate in the low and intermediate risk patients, defined as positive findings in PSMA PET/CT performed for persistence disease, may reflect the true aggressive nature of the occult disease that was missed pre-operatively. Supporting the extra-prostatic pattern of this failure, we found that local recurrence in the prostatic bed was low overall (7%) and showed no statistically significance difference between the Staging and Non-Staging Cohorts. This indicates that the observed high rate of PSA persistence in the Non-Staging Cohort was primarily driven by undetected lymphatic or distant metastatic disease, which conventional imaging failed to identify. This finding provides direct evidence that the diagnostic benefit of PSMA PET/CT is its ability to identify occult regional and distant disease, making it an indispensable tool for accurate risk stratification. A critical factor contributing to this disparity is the surgical management driven by the initial staging. Notably, 21% of patients in the Non-Staging Cohort did not undergo PLND at the time of radical prostatectomy, likely due to a low-risk assessment based on negative conventional imaging. The high rate of pelvic nodal positivity detected by PSMA PET at persistence in this group is a direct consequence of this surgical omission. Had these patients undergone pre-operative PSMA PET, the identification of occult N1 disease would have dictated a more appropriate surgical plan: specifically, mandating a curative extended eLND to remove the metastatic burden or shifting the patient towards definitive radiotherapy, thereby preventing the persistence of untreated pelvic nodal metastases. The absence of pre-operative PSMA PET/CT in the Non-Staging Cohort warrants specific clarification, particularly given the high prevalence of ISUP grade > 3 patients in this group. For patients with low to favorable-intermediate risk disease, the omission adhered to standard guidelines which do not routinely recommend molecular imaging. However, for the significant proportion of high-risk patients (ISUP 3–5), the lack of molecular staging was attributable to real-world logistical barriers prevalent during the earlier years of the study period (2019–2022), including limited scanner access and scarce availability of the PSMA radiotracer itself. Furthermore, it reflects a historical reliance on conventional imaging: as shown in our results, these patients underwent CT and bone scans which yielded negative results. This ‘false reassurance’ from conventional staging likely discouraged further investigation with PET, leading clinicians to proceed directly to radical prostatectomy despite the high-risk feature.

Moreover, while recent large cohort studies have debated the optimal timing for defining PSA persistence and its association with long-term prognosis, a key study by Tilki et al. highlighted the limitations of the conventional PSA clearance window (19). Our data support this hypothesis, providing a complementary imaging perspective, suggesting that the problem in the Non-Staging Cohort is not the delayed clearance of PSA, but rather the failure to capture significant disease burden, leading to the rapid manifestation of extensive disease at biochemical persistence. This finding supports the argument that PSMA PET’s high sensitivity for small nodal and distant sites, even at intermediate-risk levels, is essential to prevent curative local intent therapy (RP or definitive RT) from being misapplied to systemic disease (20). This highlights the inherent limitations of relying solely on pathological features for risk stratification and suggests that early PSA persistence is a powerful clinical trigger that warrants immediate molecular imaging, regardless of the initial risk group.

One of the main consequences of this diagnostic gap lies in the “theoretical change of management.” Our data suggest that a pre-operative PSMA PET/CT would have fundamentally altered the initial therapeutic strategy for all 44 positive patients in the Non-Staging Cohort. Specifically, the detection of M1 disease in 77% of these positive cases would have mandated the initial consideration of systemic therapy (e.g., intensified systemic treatments) rather than RP, avoiding an unnecessary and ineffective major surgical procedure. For the remaining 23% with extensive pelvic nodal disease, knowledge of this burden would have justified alternative aggressive local management, such as pelvic radiotherapy, long term ADT, abiraterone (21). This retrospective analysis provides evidence that PSMA PET is not just a staging tool, but a gatekeeper to appropriate therapy selection. The high rate of missed M1 disease in a cohort deemed less pathologically aggressive than the higher risk groups call for further prospective randomized research that could ultimately lead to a re-evaluation of current guidelines and a potential broadening of the indications for PSMA PET, especially for patients near the intermediate-high risk threshold who are candidates for radical local treatment.

This study is subject to several limitations inherent to its retrospective, single-center design. Firstly, the two cohorts were not randomized, exhibiting a clear selection bias where the Staging Cohort was pathologically higher-risk, consistent with current guideline recommendations. However, this inherent bias arguably strengthens our core finding, as the less pathologically aggressive Non-Staging Cohort subsequently demonstrated a higher burden of disease at persistence. Secondly, the endpoint concerning initial patient management, while compelling, remains a “theoretical change of management” based on radiological findings performed after surgery for PSA persistent disease rather than documented long-term clinical outcomes. Finally, the analysis relies solely on [68Ga]Ga-PSMA-11 PET/CT findings at the time of PSA persistence; future studies incorporating immunohistochemistry or biopsy data would further validate the pathological nature of the persistent lesions.

## Conclusion

Our analyses suggests that the omission of pre-operative [68Ga]Ga-PSMA-11 for initial staging is associated with a significantly higher incidence of extensive PSMA-positive disease (both advanced locoregional and distant metastatic) at the time of PSA persistence. This study highlights the clinical cost of the under-staging gap, revealing that patients who bypassed molecular imaging were more likely to harbor unrecognized M1 disease, fundamentally misguiding their initial therapeutic path towards ineffective local therapy. These findings support the vital role of early molecular imaging in achieving accurate risk stratification and optimizing treatment planning,, particularly for those on the borderline of intermediate-to-high risk, to ensure appropriate triage to either local or systemic treatment.

## Data Availability

The datasets generated during and/or analyzed during the current study are available from the corresponding author for reasonable requests. This study was performed in line with the principles of the Declaration of Helsinki. This study has been approved by the institutional review board (Ethics Committee of IRCCS Azienda Ospedaliero-Unversitaria Policlinico S. Orsola di Bologna (244/2016/O/Oss—8 November 2016). All subjects/patients/participants signed an informed consent form.

## Declarations

The authors declare that no funds, grants, or other support were received during the preparation of this manuscript.
